# Study on the use of black phosphorus quantum dots in the treatment of atherosclerosis

**DOI:** 10.18632/aging.206205

**Published:** 2025-02-20

**Authors:** Shengwei Zhang, Yiran Ji, Bingxuan Xu, Die Hu, Xue Zhang, Yujian Song, Keke Chen, Yilin Wen, Xiaoxin He, Yun Chen, Tingting Zheng

**Affiliations:** 1 Shenzhen Key Laboratory for Drug Addiction and Medication Safety, Department of Ultrasound, Institute of Ultrasonic Medicine, Peking University Shenzhen Hospital, Shenzhen Peking University, The Hong Kong University of Science and Technology Medical Center, Shenzhen 518036, Guangdong, P.R. China; 2 Department of Ultrasound, Xiaolan People’s Hospital of Zhongshan, Zhongshan 528415, Guangdong, P.R. China; 3 Ultrasound Diagnosis and Treatment Center of the First People’s Hospital of Foshan, Foshan 528000, Guangdong, P.R. China; 4 Department of Ultrasound, Nanjing Drum Tower Hospital, Nanjing 210000, Jiangsu, P.R. China

**Keywords:** atherosclerosis, black phosphorus quantum dots, plaque clearance, vascular elasticity, ultrasonic imaging evaluation

## Abstract

Atherosclerosis is the pathological basis of cardiovascular disease, and there are no clinical drugs that can safely and efficiently remove atherosclerotic plaques. In this study, black phosphorus quantum dots (BPQDs) were applied to the treatment of atherosclerosis in high fat diet ApoE^-/-^ model mice that BPQDs were given every other day for 3 weeks without changing the high-fat diet. 45.3% atherosclerotic plaque was cleared efficiently within 3 weeks in BPQDs intravenous administration way every other day. The treatment was more effective than traditional statins. The findings suggest that BPQDs have great potential to be applied for clinical prevention and treatment of AS that does not require dietary changes.

## INTRODUCTION

Cardiovascular disease (CVD) is the leading cause of death worldwide, according to the latest authoritative statistics. Moreover, coronary heart disease (CHD) is the leading cause of CVD death, followed by stroke, both of which are closely related to the pathological changes of atherosclerosis [1]. The pathology of atherosclerosis has been described as a diffuse and progressive process, with a variable distribution and clinical presentation, which is dependent on the regional circulation involved [2]. Atherosclerosis in coronary and cerebral arteries leads to stenosis or blockage of corresponding arteries, which is the direct cause of CHD and stroke. As a chronic inflammatory disease, atherosclerosis has been demonstrated to be accelerated or inhibited by immune mechanisms [3, 4]. Inflammation plays a central role in atherosclerosis and develops concurrently with the accumulation of minimally oxidized low-density lipoprotein (ox-LDL) in the arterial wall. In the intima, LDL undergoes oxidative modification by reactive oxygen species (ROS), which promotes the uptake of lipids into macrophages [5]. Macrophages represent a major cell type in early atherosclerotic lesions and play important roles at all stages of lesion progression. The phenotype of macrophages in atherosclerotic lesions is likely influenced by both lineage commitment and phenotypic changes in response to their environment. However, macrophages in atherosclerotic arteries eventually become lipid-laden foam cells through a process regulated by the balance between the uptake of modified LDL and the efflux of cholesterol and other lipids [6, 7]. Thus, pharmacological regulation of macrophage phenotypes and reduction of foam cells represent promising therapeutic strategies for atherosclerosis.

Developments in nanotechnology have led to nanomedicine, which focuses on the design, manufacture, and characterization of nanomaterials for the delivery of drugs and prevention, diagnosis, and treatment of diseases [8]. The therapeutic delivery and imaging mediated by nanomaterials are superior to those of the traditional systemically free drug and contrast agent delivery. The properties of nanomaterials are highly controllable because their physical properties and chemical compositions can be easily adjusted to achieve specific functions. Nanomaterials are expected to offer great opportunities for advancing the diagnosis and treatment of atherosclerosis [9].

Black phosphorus (BP) is an emerging nanomaterial that has recently entered the biomedical field [10, 11]. Black phosphorus quantum dots (BPQDs), as allotropes of the common element phosphorus in organic organisms, have been used in the diagnosis and treatment of a variety of inflammation-related diseases in recent years due to their superior biocompatibility and great medical application potential [12, 13]. Many important applications of BPQDs have been explored in succession, covering a broad range of fields, especially bioimaging, fluorescence sensing, nonlinear optical absorbers, cancer therapy, intelligent electronic elements, photovoltaics, optoelectronics, and flexible devices [14, 15]. Regulation of macrophage phenotypes and promotion of autophagy by BPQDs have also been reported [16]. However, BPQDs have been mainly applied to cancer therapy studies, and there are currently no reports on its application in the treatment of atherosclerosis.

**Figure 1:**
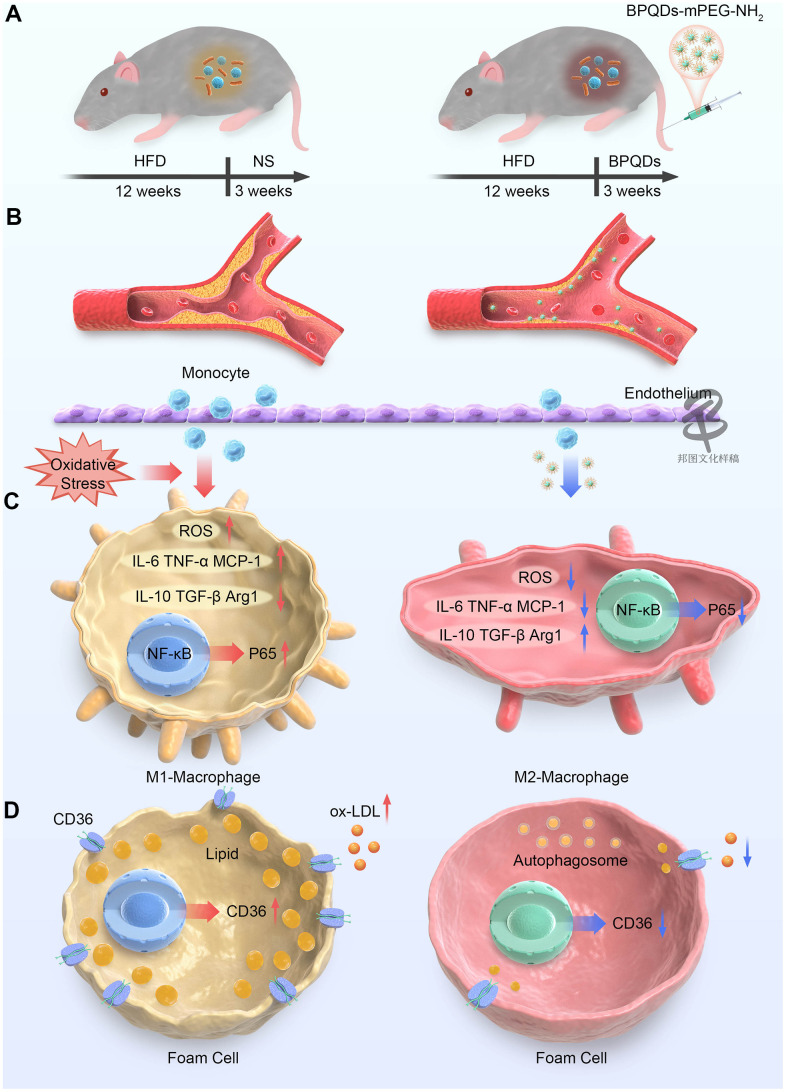
**Schematic representation of BPQDs treating atherosclerosis in mice.** (**A**) Schematic diagram of changes in bacterial flora of atherosclerotic mice before and after BPQDs treatment. (**B**) Schematic diagram of atherosclerotic plaques before and after BPQDs treatment. (**C**) Schematic diagram of changes in cell phenotype of macrophages before and after BPQDs treatment. (**D**) Schematic diagram of changes in foam cells before and after BPQDs treatment. (HFD: high-fat diet, NS: normal saline, BPQDs: black scale quantum dots, MPEG-NH 2: aminopolyethylene glycol, ROS: Reactive oxygen species, IL-6: interleukin-6, TNF-α: tumor necrosis factor-α, MCP-1: monocyte chemoattractant protein-1, IL-10: interleukin-10, TGF-β: transforming growth factor β, Arg1: type 1 arginase).

Based on the effects of regulating macrophage phenotypes and promoting autophagy, we expect BPQDs to be a promising method to reduce atherosclerosis. In this study, we applied BPQDs to the treatment of atherosclerosis in mice to investigate their ability to eliminate atherosclerotic plaques and restore aortic vascular elasticity, before further exploring the possible mechanisms (Figure 1).

## MATERIALS AND METHODS

### Modification and characterization of BPQDs

Experimental materials and sources are shown in Supplementary Table 1.

The dispersion solution of BPQDs (concentration 0.5 mg/ml) stored at 4° C was removed, and after ultrasonic shaking was uniform (30s), 200 μl of BPQDs dispersion was sucked into a 1.5 ml EP tube using a pipette gun. The EP tube was placed in a centrifuge, balanced, and centrifuged (centrifugation at 12500 rpm for 18 min). After the completion of the centrifugal supernatant, to join 200 mu l amino polyethylene glycol (mPEG2000 - NH_2_) solution concentration (1 mg/ml), ultrasonic oscillation even after (2 min), modified 1 h at room temperature, centrifuge again 12500 rpm (centrifugal conditions, 18 min). After centrifugation, the supernatant was discarded, 1 ml normal saline was added, and the modified BPQDs in normal saline solution (concentration 0.1 mg/ml) was obtained after ultrasonic shaking (2 min).

The particle size and zeta potential of BPQDs before and after modification were measured by DLS, and the morphology of BPQDs before and after modification was photographed by transmission electron microscopy (TEM). The pegylated BPQDs were suitable for subsequent experiments.

### Safety evaluation of BPQDs

Macrophages are not only systemic inflammatory cells, but also key cells in the occurrence and development of atherosclerosis, which is a hot research spot in the field of atherosclerosis [17]. Macrophages (RAW264.7) were further used to explore the effect and mechanism of BPQDs on cells.

### Cell safety

Macrophages and aortic endothelial cells play important roles in the occurrence and development of atherosclerosis. Here, RAW264.7 macrophages and mouse aortic endothelial cells (MAECs) were used to verify the cell safety of BPQDs. The viability of RAW264.7 macrophages and MAECs under gradient concentration of BPQDs was detected by cell counting kit-8 (CCK-8).

### In vivo safety

Apoe^−/−^ mice develop atherosclerosis with a high-fat diet and are commonly used as an animal model for studying atherosclerosis, with C57BL mice representing their normal wild-type counterpart [18]. In this study, C57BL mice were divided into two groups (n = 5), comprising experimental and control groups, which were intravenously injected through the caudal vein with 0.1 mL normal saline and 0.1 mL BPQDs in normal saline solution (0.1 mg/mL), respectively, for a course of 3 weeks, with 3 injections/week (Supplementary Table 2). After completing the course, blood was collected from the inferior vena cava for serum detection after the mice were placed under abdominal anesthesia, and the heart, liver, and kidney were collected to generate pathological sections.

### Mechanism of BPQD action on cells

### Regulation of ROS

RAW264.7 macrophages were induced into an inflammatory state by lipopolysaccharide (LPS), before treating with BPQDs and measuring the ROS expression in each group (control, model, BPQDs) by laser scanning confocal microscopy and flow cytometry.

### Regulation of macrophage polarization

Following the protocol outlined in 2.3, the cells of each group were collected to extract protein and RNA. The protein was used for western blotting to detect the expression of the NF-κB signaling pathway regulatory factor P65, and the RNA was used for real-time quantitative PCR to detect the gene expression of cytokines and chemokines.

### Regulation of lipid metabolism

RAW264.7 macrophages were induced to foam cells by ox-LDL before treatment with BPQDs, staining with oil red O staining in each group (control, ox-LDL, BPQDs), and detection using an optical microscope. Phagocytosis of macrophages on ox-LDL labeled by fluorescein was detected by fluorescence microscopy. The cells of each group were collected to extract protein and RNA. The protein was used for western blot to detect the expression of the cell surface scavenger receptor CD36 and the autophagy marker protein LC3-II/I, while the RNA was used for real-time quantitative PCR to detect the gene expression of CD36 and other lipid metabolic factors.

### Effect of BPQDs on atherosclerotic model mice

An overview of the mouse modeling and treatment are shown in Table 1.

**Table 1:** Grouping and administration of mice.

**Group**	**Mice (n≥10)**	**Diets**	**Dosage**	**Frequency**
Control	C57BL	Normal diet	0.1 mL normal saline	3 times/week
Model	ApoE^−/−^	High fat diet	0.1 mL normal saline	3 times/week
BPQDs	ApoE^−/−^	High fat diet	0.1 mL BPQDs normal saline solution	3 times/week

### Ultrasonography

The aortic plaque, cardiac function, inner diameter, and velocity of large vessels were detected by small animal ultrasonography before and after treatment.

Arterial vascular elasticity = (ESD − EDD)/ESD * 100%.

(ESD: End-systolic diameter, EDD: End-diastolic diameter)

### Photoacoustic microscope

The abdominal wall of mice was cut open under gas anesthesia. The surrounding tissue of the abdominal aorta was carefully separated, and part of the abdominal aorta was dissociated with a thin flexible tube. The changes in the internal diameter of the abdominal aorta were monitored with a photoacoustic microscope at the dissociative abdominal aorta. After 5 min of monitoring, 0.1 mL quinidine solution was injected into the mouse caudal vein, and the internal diameter of the abdominal aorta was monitored by a photoacoustic microscope at the same dissociative part of the abdominal aorta. The model and parameters of the photoacoustic microscope are shown in Supplementary Table 2.

### In vitro experiment

After placing mice under abdominal anesthesia, blood was collected from the inferior vena cava to detect the levels of blood glucose, serum inflammatory factors, and other indicators. Fresh feces were collected from mice for intestinal flora detection. After cardiac perfusion with phosphate buffered saline (PBS), the heart, liver, and kidney were taken for pathological section examination, and the aorta was taken for oil red O staining to calculate the plaque area. A small part of the aortic arch, innominate artery, and abdominal aorta were taken for TEM.

## RESULTS AND DISCUSSION

### Preparation and characterization of BPQDs

**Figure 2:**
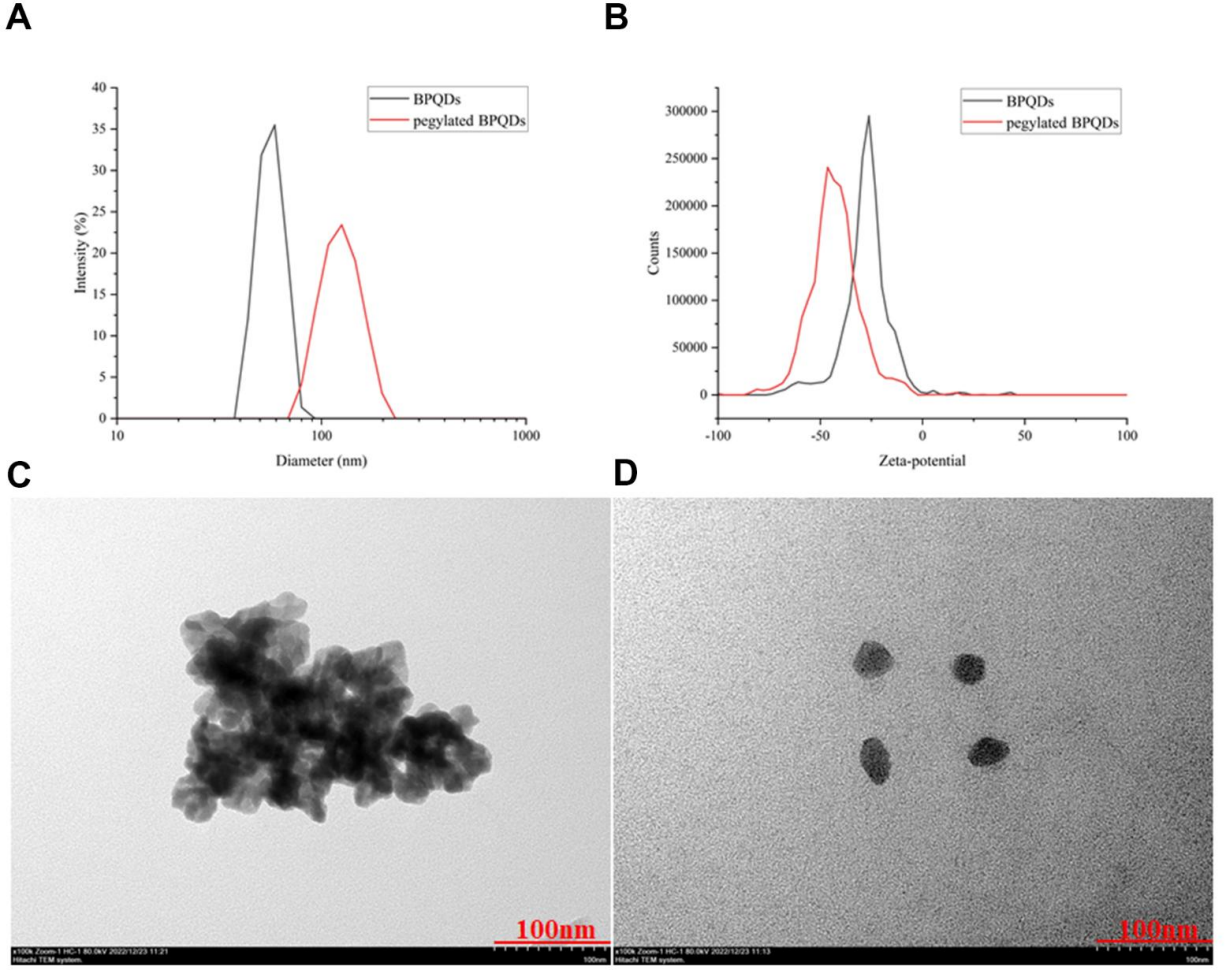
(**A**) Comparison of hydrated diameters of BPQDs after filtration in black, while pegylated BPQDs in red. (**B**) Zeta potential comparison of BPQDs before and after PEG2000 modification. The black solid curve represents the zeta potential distribution before modification, and the red solid curve represents the zeta potential distribution after modification. (**C**) 2D BPQDs tend to self-stack by π-π stacking before hydrophilic chain PEG2000 modification. (**D**) Pegylated BPQDs no longer agglomerated, and the stability of the emulsion was enhanced, which tended to be free to disperse.

The particle size, zeta-potential, and morphology before and after modification of BPQDs by PEG were determined by transmission electron microscope (TEM) and are shown in Figure 2. The particle size of BPQDs increased after PEG-based modification, from a mean of 60 nm to 105 nm, demonstrating successful modification of BPQDs by PEG (Figure 2A). After PEG-based modification, the zeta potential value of BPQDs decreased from a mean of −25 to −50, demonstrating that the stability of BPQDs in solution improved after modification (Figure 2B). Moreover, the form of BPQDs was aggregated into a group before modification, while the form after modification was a single sheet, indicating that BPQDs modified by PEG were more easily dispersed and their chemical properties were more stable (Figure 2C, Figure 2D).

In DLS results, the hydration particle sizes of BPQDs and pegylated BPQDs were determined immediately after ultrasonic oscillation, so their size by DLS showed that BPQDs was smaller than pegylated BPQDs. However, in the results of TEM, although we also started the experiment immediately after the dispersion of BPQDs and pegylated BPQDs by ultrasonic oscillation, the experimental procedures of TEM are more complicated. After these complicated experimental procedures, BPQDs aggregated into a group while pegylated BPQDs still dispersed well as single sheet in TEM images. The results of the two experiments show that pegylated BPQDs have better dispersion and stability than BPQDs.

In addition, the particle size distribution and zeta potential of BPDQs in different temperatures and conditions (LPS and ox-LDL) were shown in supporting information (Supplementary Figure 1). The results show that different temperatures and conditions (LPS and ox-LDL) have no significant effect on the particle size distribution and zeta potential of BPDQs. How black phosphorus degradation and metabolism occur after intravenous administration was not involved in this study, but it has been studied in related reports [19].

### Safety evaluation of BPQDs

**Figure 3:**
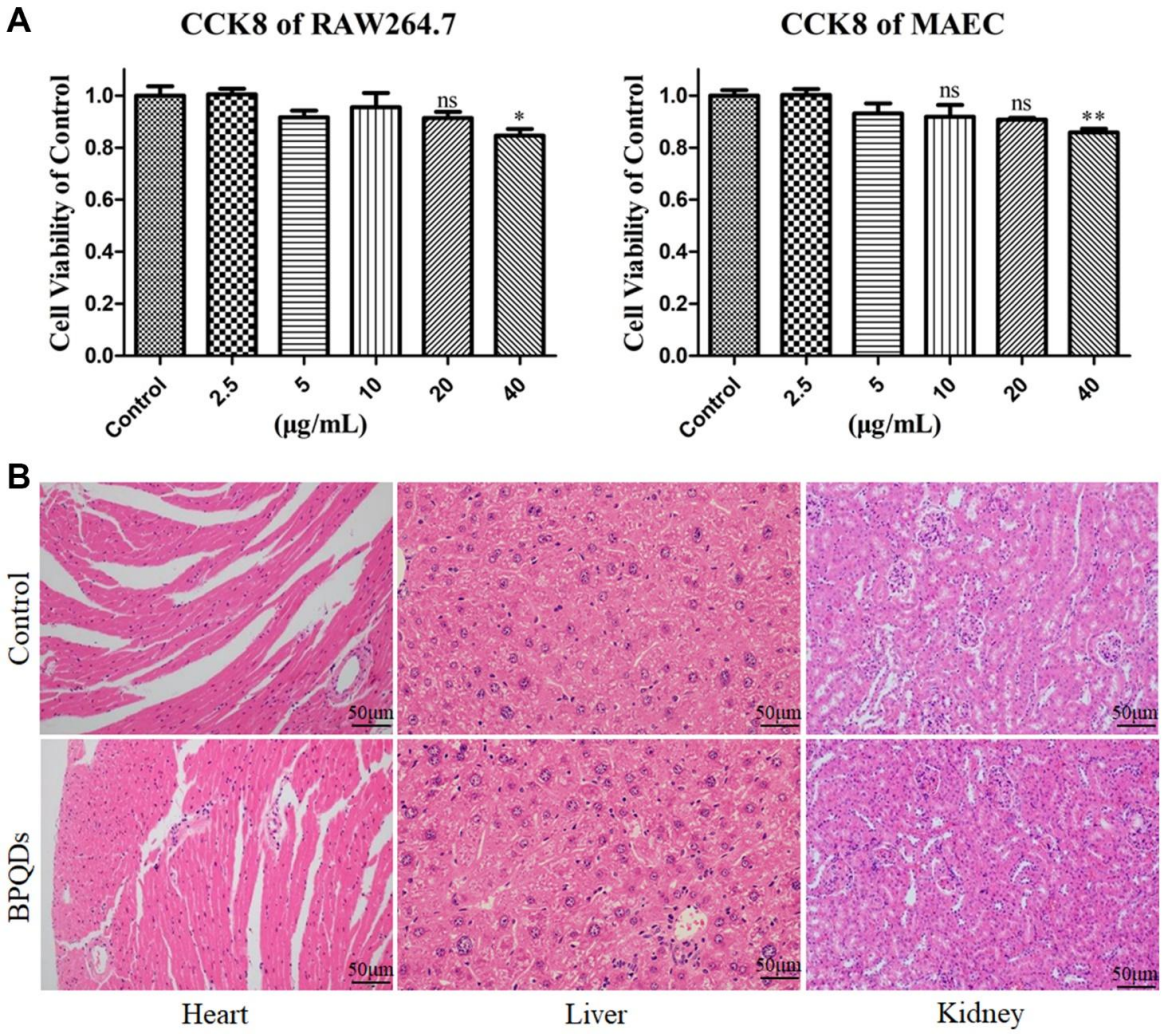
(**A**) Viabilities of RAW264.7 macrophages and MAECs after treatment with gradient concentrations of BPQDs. (**B**) Pathological sections of the heart, liver and kidney.

The results of CCK-8 assay showed that the low concentration of BPQDs had no obvious toxic effect on macrophages and endothelial cells, while 20 μg/mL was the optimal concentration, which was used in the following cell experiments (Figure 3A). Pathological sections of mice showed no significant difference in the tissue structure of heart, liver, and kidney between the control and BPQD groups (Figure 3B). Serum detection showed no significant difference in the serum oxidation factor levels between the control and BPQD groups as well as body weight of mice (Supplementary Figure 2). The results indicated that BPQDs showed no obvious toxicity to the heart, liver, and kidney, and did not cause an obvious oxidative stress response in mice. The results of cytotoxicity and animal experiments suggest that BPQDs were safe *in vivo* and *in vitro*.

BPQDs are allotropes of phosphorus with active chemical properties which are necessary to be stabilized to apply them in biomedicine. Several methods can be used to stabilize the chemical properties of BPQDs [14]; these include BPQD/nanosheet hybrid, BPQD composite film, BPQD molecular complex, BPQD self-assembly, and BPQD polymer modification [20–22]. Among these functionalization modification methods, BPQD polymer modification is more suitable for BPQDs application in the biomedical field. BPQDs are soluble but easily aggregated in water or salt solution. PEG has high biocompatibility and is an excellent polymer stabilizer for nanomaterials in biomedical applications. The combination of BPQDs with PEG is a good method to improve their stability in physiological media. Positively charged PEG-NH_2_ is coated on the surface of BPQDs by electrostatic adsorption. The pegylated BPQDs show low biotoxicity to various cell types [12]. By coupling with PEG, BPQDs retain a high photothermal conversion performance and low degradability, as well as improved biocompatibility and physiological stability, after 48 h incubation in aqueous solution [22]. BPQD modification by polymer PEG was adopted as the functional modification in this study, and the resulting BPQDs showed higher dispersion and stability than BPQDs in physiological solutions.

### Mechanism of BPQD action on cells

### Regulation of ROS

**Figure 4:**
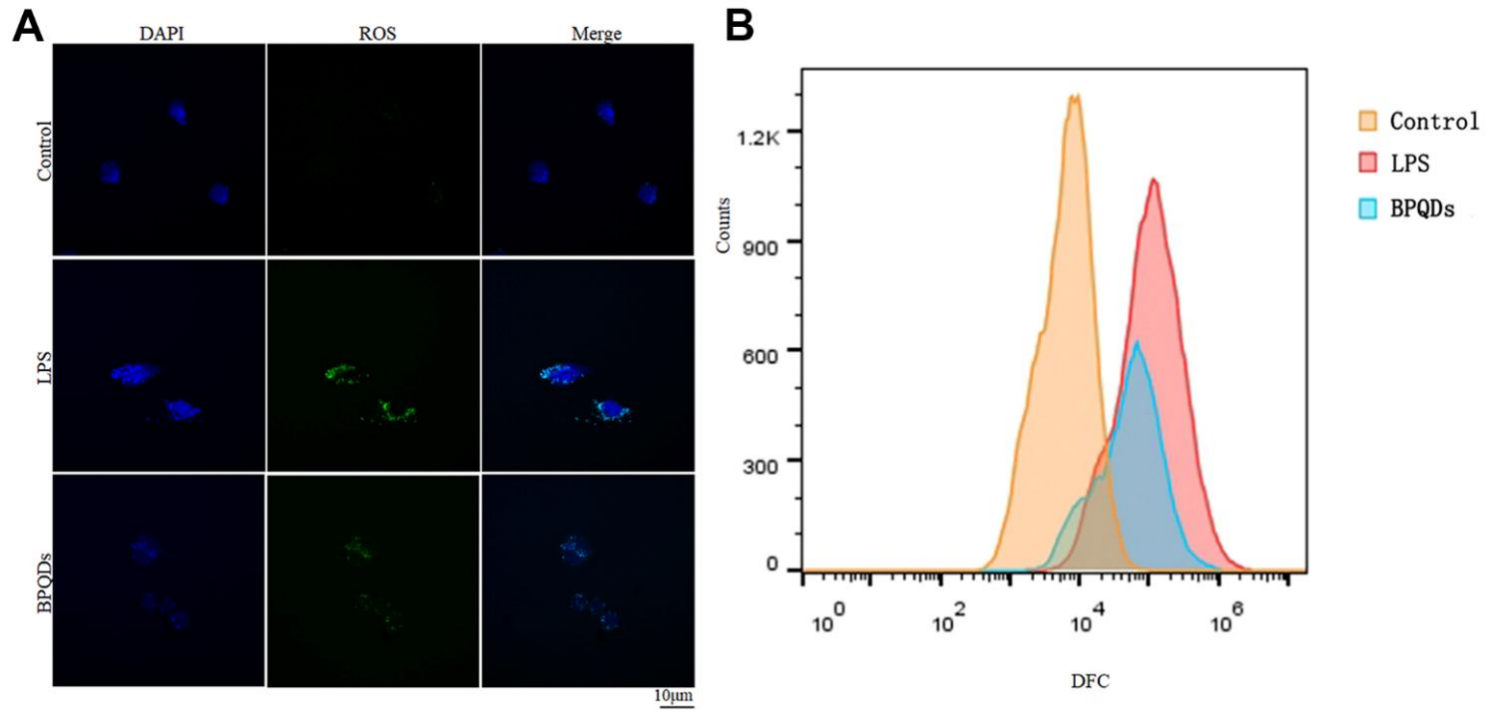
(**A**) ROS fluorescence intensity observed by confocal. (**B**) Flow cytometry of fluorescence-labeled cell counts.

Confocal images showed that the ROS fluorescence intensity in the BPQD group was significantly lower than that in the LPS group (Figure 4A and Supplementary Figure 3). Moreover, the results of flow cytometry showed that the count of macrophages with ROS fluorescence expression in the BPQD group was significantly lower than that in the LPS group (Figure 4B and Supplementary Figure 3). The results show that BPQDs reduced the ROS level of macrophages and regulated the oxidative stress response.

Oxidative stress plays critical functions in the biology of macrophages throughout different stages of atherogenesis. ROS play a central role in most processes and pathophysiology of atherosclerosis by regulating monocyte priming, adhesion, and recruitment, as well as macrophage differentiation, activation, polarization, death, and autophagy [23]. The amount of ROS production can often be used to judge the severity of oxidative stress and inflammation. Our results showed that BPQDs reduced ROS at the cellular level but had no significant impact on the serum oxidation factor levels of mice in the *in vivo* safety evaluation. These results indicate that the ROS reduction effect of BPQDs may have relatively high targeting to macrophages, which act at the cellular level without causing significant serological changes in mice. The targeting and mechanism of ROS reduction of BPQDs are worthy of further study in the future.

### Regulation of macrophage polarization

**Figure 5:**
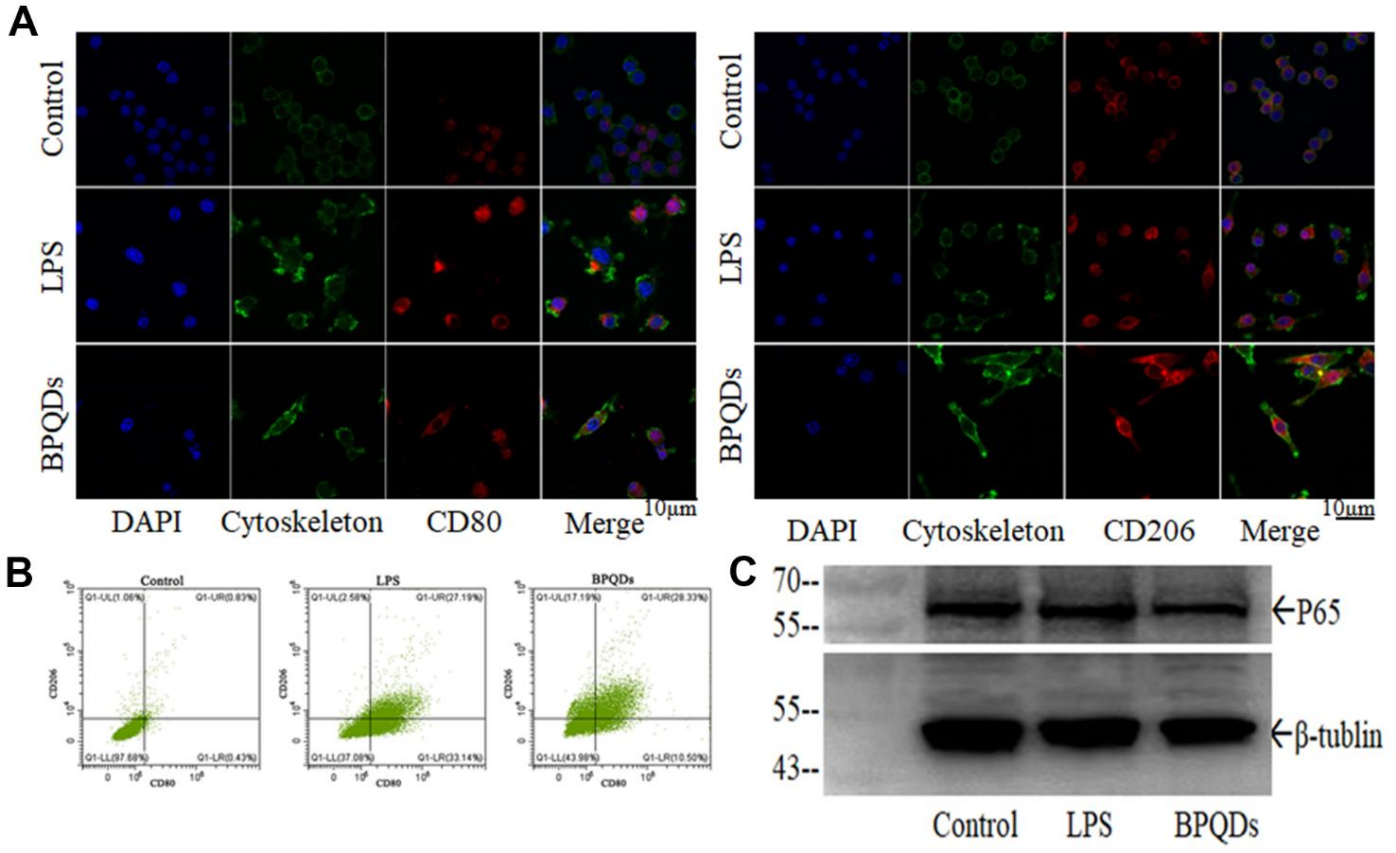
(**A**) Fluorescence intensity of CD80 and CD206 in macrophages observed by confocal. (**B**) Flow cytometry of macrophages expressing CD80 and CD206. (**C**) Western blot of P65.

Confocal images showed that the fluorescence intensity of CD80 in the LPS group significantly increased, while that of CD206 was not. The fluorescence intensity of CD80 in the BPQD group decreased, while that of CD206 increased compared to the LPS group. In parallel, in terms of cellular morphology, the confocal images showed that the macrophages of the LPS group had antennae, while those of the BPQD group had spindle morphology, which happens to be the typical morphology of M1 polarized and M2 polarized macrophages, respectively (Figure 5A and Supplementary Figure 4A). Flow cytometry showed that the proportion of macrophages expressing CD80 in the LPS group significantly increased, while the proportion of macrophages expressing CD206 in the LPS group slightly increased. The proportion of macrophages expressing CD80 in the BPQD group significantly decreased compared to that in the LPS group, while the proportion of macrophages expressing CD206 was significantly higher than that in the LPS group (Figure 5B and Supplementary Figure 4B). The results indicate that BPQDs inhibited M1 polarization but promoted M2 polarization of macrophages.

Western blot showed that the expression level of P65 in the BPQD group was lower than that in the LPS group (Figure 5C and Supplementary Figure 4C). Real-time quantitative PCR showed that the gene expression levels of pro-inflammatory cytokines and chemokines in macrophages in the LPS group increased, while those in the BPQD group decreased. Furthermore, the gene expression levels of inhibitory cytokines increased in the BPQD group, but not in the LPS and control groups (Supplementary Figure 5A, 5B). The results indicated that BPQDs reduced the expression of regulatory factor P65 and had a regulatory effect on NF-κB signaling pathway, inhibiting M1 macrophages polarization but promoting M2 polarization. Moreover, BPQDs reduced the gene expression of M1-polarized pro-inflammatory cytokines and chemokines in macrophages while increasing the gene expression of M2-polarized inhibitory cytokines in macrophages, thereby playing a role in regulating inflammation.

Transcriptional and epigenetic adaptation within immune cells play an important role in the pathogenesis of atherosclerosis [24]. Different macrophage phenotypes allow them to engulf lipids, dead cells, and other substances perceived as danger signals; efflux cholesterol to high-density lipoprotein (HDL); proliferate and migrate; undergo apoptosis and death; and secrete numerous inflammatory and pro-resolving molecules [25]. Macrophages have two main phenotypes: M1 and M2. M1 macrophages are considered classically activated macrophages, which secrete pro-inflammatory cytokines and chemokines, are adept at presenting antigens, participate in the positive immune response, and function in immune surveillance. In contrast, M2 macrophages are vicariously activated and mainly secrete inhibitory cytokines to downregulate the immune response [26, 27]. Currently, CD80 is generally considered to be the superficial marker protein of M1-polarized macrophages, while CD206 is the superficial marker protein of M2-polarized macrophages [28, 29]. LPS-induced macrophage inflammation activates the NF-κB signaling pathway, which is one of the classical signaling pathways regulating macrophage polarization [30]. NF-κB is the primary transcription factor involved in the cellular response to harmful stimuli, and regulatory factor P65 is the most common dimer of NF-κB [31]. In *in vitro* experiments, we demonstrated that BPQDs promoted the polarization of macrophages from M0 to M2 and inhibited the polarization from M0 to M1, thus reducing the expression of pro-inflammatory factors and reducing the inflammatory response.

### Regulation of lipid metabolism

**Figure 6:**
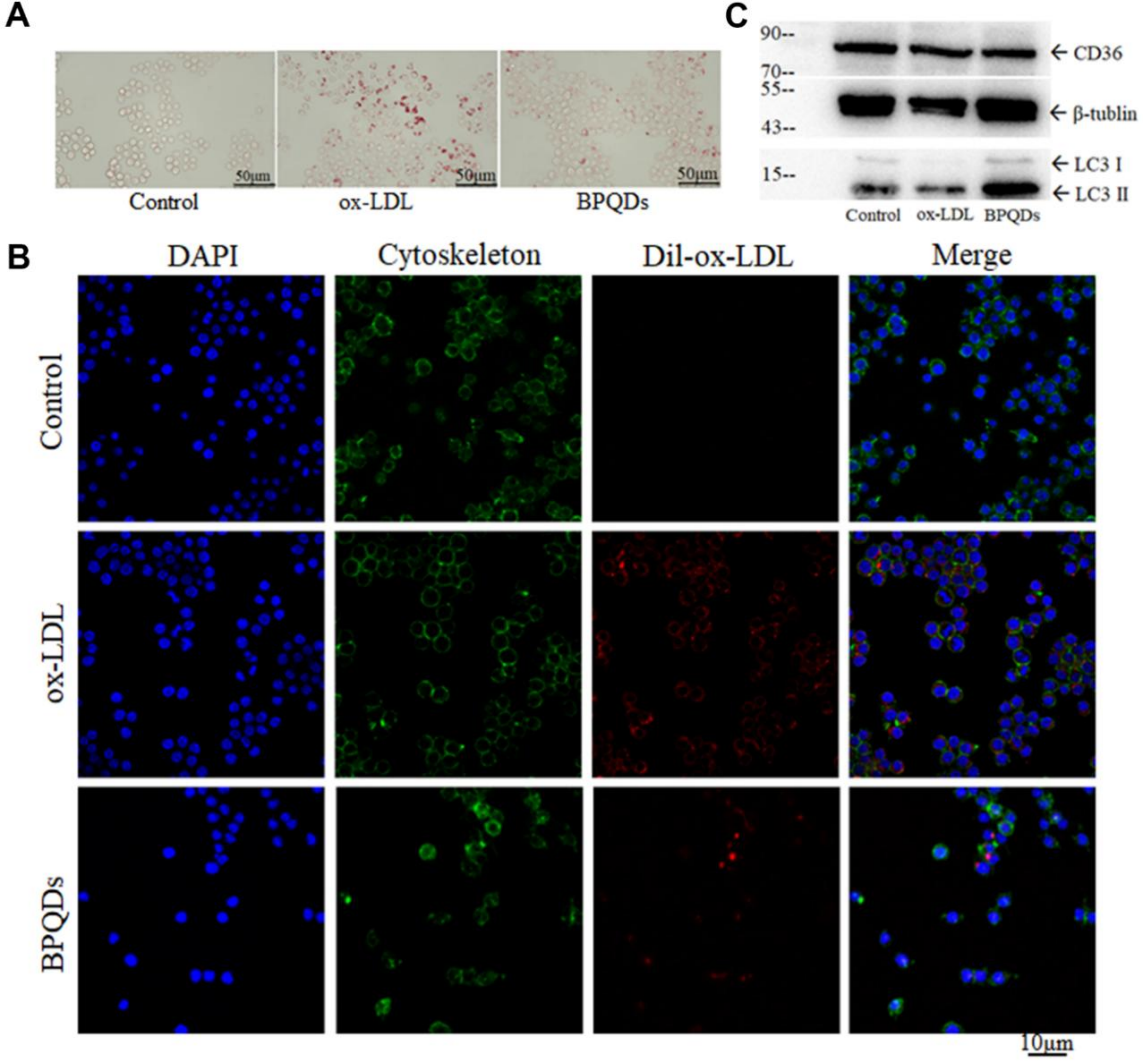
(**A**) Oil red O staining of macrophages/foam cells. (**B**) Fluorescence intensity of ox-LDL observed by confocal. (**C**) Western blot of CD36 and LC3 II / I.

Oil red O staining images showed that the stained area of the ox-LDL group increased, while that of the BPQD group decreased compared to that of the ox-LDL group (Figure 6A and Supplementary Figure 6A). Confocal images showed increased fluorescence intensity in the ox-LDL group, while that in the BPQD group decreased (Figure 6B and Supplementary Figure 6B). The results indicate that macrophages phagocytize ox-LDL and evolve into foam cells, and that BPQDs reduced the uptake of ox-LDL by macrophages.

Western blot showed that the expression level of CD36 in the BPQD group was lower than that in the ox-LDL group (Figure 6C and Supplementary Figure 6C). The ratio of LC3-II/I in the BPQD group was higher than that in the ox-LDL group (Figure 6C and Supplementary Figure 6D). Real-time quantitative PCR showed that the gene expressions of factors that promote the entry of lipids into macrophages or inhibit the lipid metabolism of macrophages increased in the ox-LDL group, while that in the BPQD group decreased compared to the ox-LDL group. The gene expressions of factors that inhibit the entry of lipids into macrophages or promote the lipid metabolism of macrophages decreased in the ox-LDL group, while that in the BPQD group increased compared to the ox-LDL group (Supplementary Figure 7).

The results indicated that BPQDs decreased the expression of the lipoprotein receptor CD36 at both gene and protein expression levels. BPQDs may regulate the gene expression of various lipid metabolism factors to reduce the formation and accumulation of foam cells. The increase of the LC3-II/I ratio suggested that BPQDs promoted autophagy of macrophages.

Abnormal lipid metabolism is the most important risk factor in atherosclerosis and CVD [32, 33]. Macrophages gather in the vessel wall and phagocytose lipids, before gradually evolving into foam cells, representing a key step in the development of atherosclerosis. Foam cells accumulate in the vessel wall and undergo fibrosis and calcification, eventually forming atherosclerotic plaques [34]. Reducing the formation and accelerating the removal of foam cells is an important idea in the treatment of atherosclerosis. CD36 is a type 2 cell surface scavenger receptor that is widely expressed in many immune and non-immune cells. It functions as both a signaling receptor responding to danger-associated molecular patterns (DAMPs) or pathogen-associated molecular patterns (PAMPs), as well as being a long chain free fatty acid transporter [35]. Macrophage CD36 participates in atherosclerotic arterial lesion formation through its interaction with ox-LDL, which triggers signaling cascades for inflammatory responses [36]. Reducing CD36 expression in cells will help to reduce the uptake of ox-LDL by macrophages and thus reduce foam cell formation. Through *in vitro* experiments, we found that BPQDs inhibited the uptake of ox-LDL by macrophages, thus inhibiting the transformation of macrophages into foam cells.

Apoptosis and autophagy, two types of programmed cell death, influence the development and progression of atherosclerosis via the modulation of cells [37]. Autophagy is a reparative, life-sustaining process by which cytoplasmic components are sequestered in double-membrane vesicles and degraded upon fusion with lysosomal compartments [38]. However, when macrophages phagocytose large amounts of lipids and evolve into foam cells, they lose many functions, including autophagy. As foam cells cannot clear themselves without the function of autophagy, they accumulate in the walls of vessels, and eventually become the main cellular component of atherosclerotic plaques [39]. Microtubule-associated protein light chain 3 is an ubiquitin-like protein that is essential for autophagy. The lipidation reaction leads to a conformational change in LC3 that is critical in autophagosome formation [40]. Because the relative amount of LC3-II/I reflects the abundance of autophagosomes, the LC3-II/I isoform is currently the most widely used molecular marker to detect autophagosomes. Through *in vitro* experiments, we found that BPQDs promote autophagy of foam cells, thus reducing their accumulation.

### Therapeutic effects of BPQDs on atherosclerotic model mice

An overview of the mouse modeling and treatment are shown in Table 1.

### Ultrasonography

**Figure 7:**
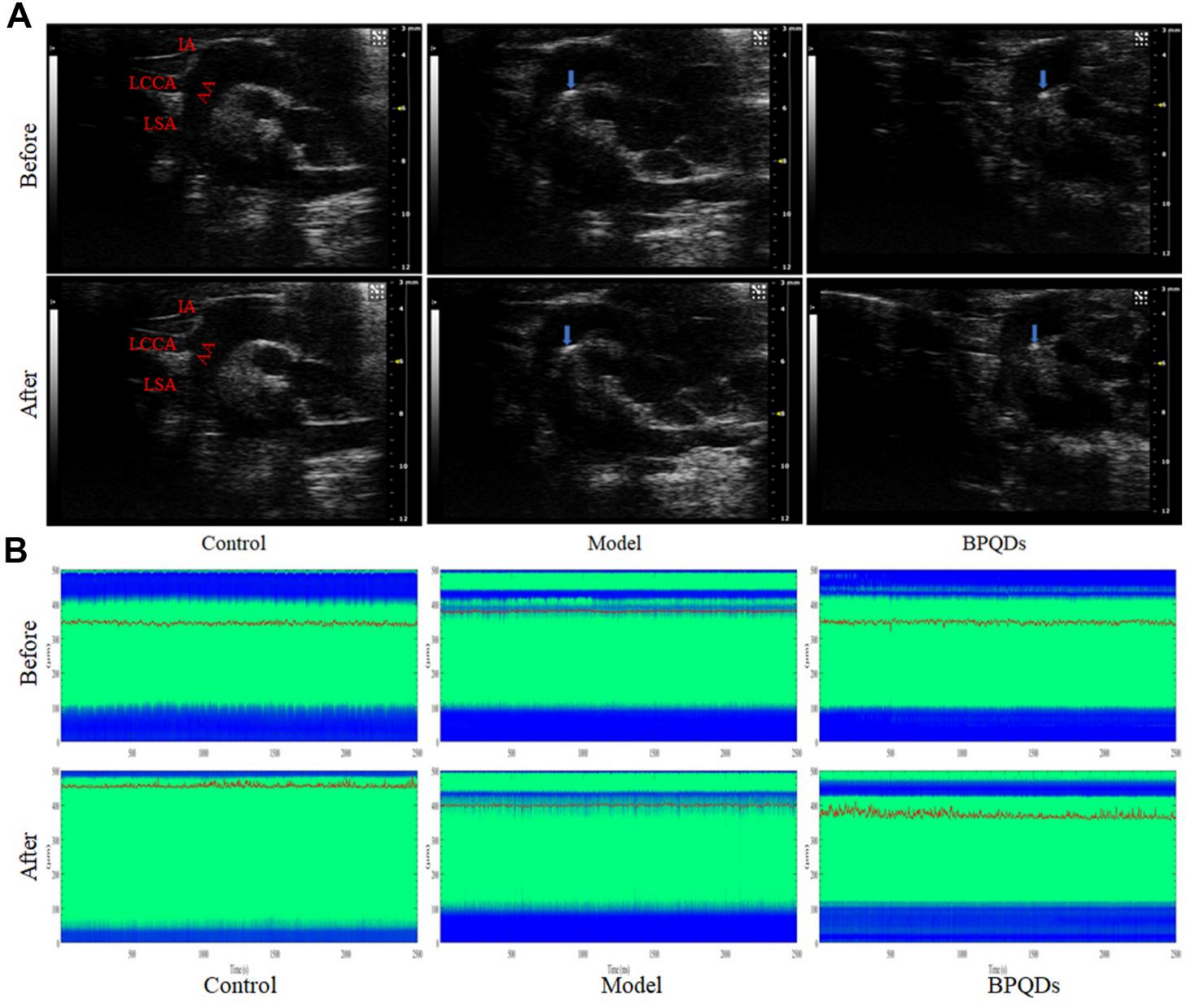
(**A**) Ultrasonography of the aortic plaque (blue arrow) before and after treatment. AA: Aortic arch, IA: Innominate artery, LCCA: Left common carotid artery, LSA: Left subclavian artery. (**B**) Comparison of photoacoustic microscopy images of abdominal aorta in mice after treatment. (Before: before the injection of vasodilator, After: after the injection of vasodilator, the green area between the blue bands indicates the internal diameter of the abdominal aorta, the red curve shows the change of the internal diameter of the abdominal aorta over time, and the corresponding ordinate is the internal diameter of the abdominal aorta).

Ultrasonography showed that atherosclerotic plaques appeared in the aortic arch of Apoe^−/−^ mice fed a high-fat diet for 12 weeks (including the model group and BPQD group), demonstrating successful establishment of the atherosclerotic mouse model. After 3 weeks of treatment, the plaques in the aortic arch of the model group continued to grow, while that in the BPQD group decreased (Figure 7A). The innominate artery elasticity of the BPQD group was higher than that of the model group, and the peak systolic velocity (PSV) of the innominate artery in the BPQD group was higher than that in the model group (Supplementary Figure 8A, 8B).

### Photoacoustic microscope

Photoacoustic microscopy showed that the abdominal aortic vascular elasticity in the BPQD group was higher than that in the model group, and the response of the abdominal aorta to the vasodilator quinidine in the BPQD group was higher than that in the model group (Figure 7B and Supplementary Figure 8C).

### Results of the in vivo experiment

**Figure 8:**
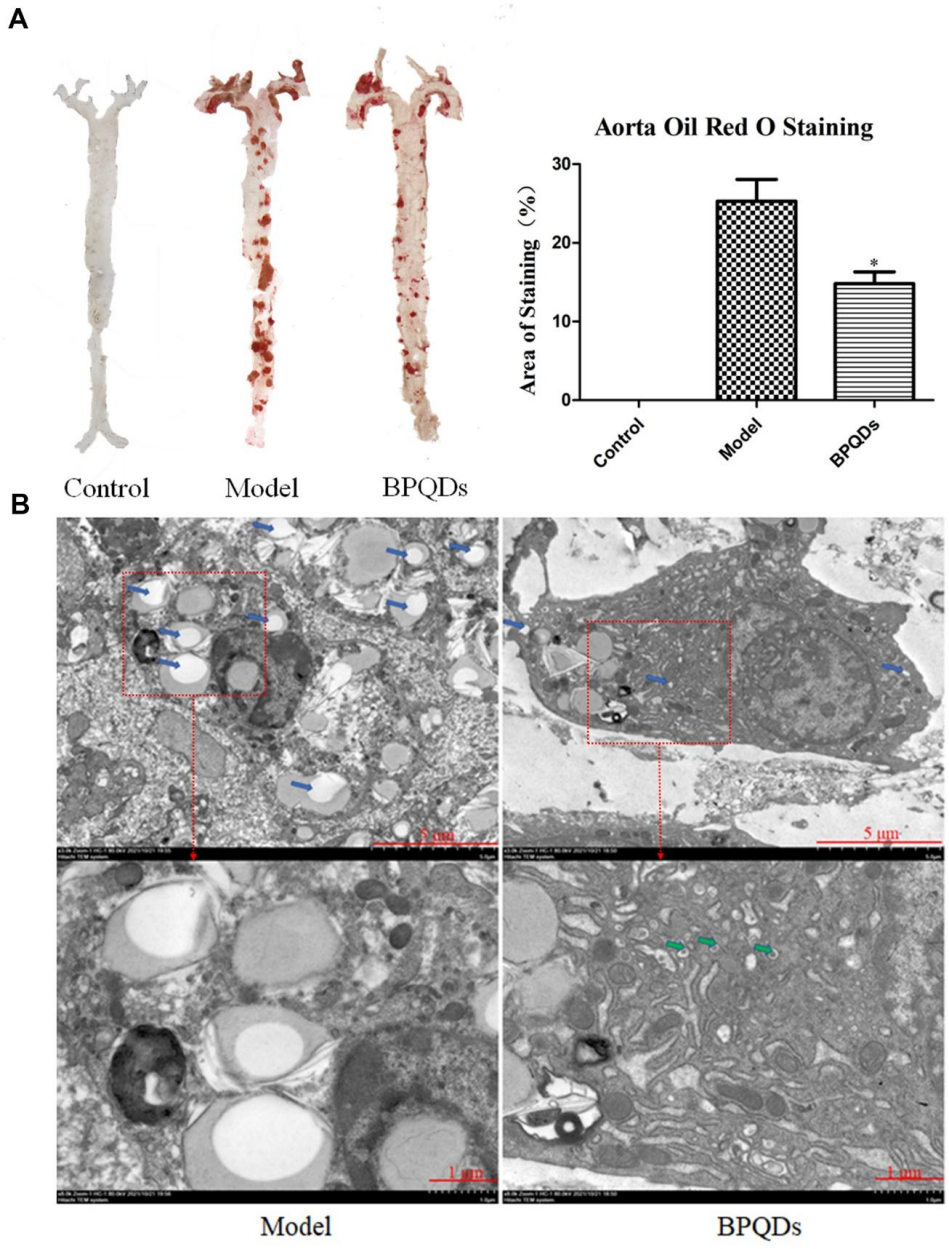
(**A**) Comparison and quantification of oil red O staining photos of aorta of mice after BPQDs treatment. (**B**) Comparison of electron microscopic photographs of aorta of mice treated with BPQDs. (Blue arrow: lipid droplet, green arrow: autophagosome).

Aorta oil red O staining showed a large area of plaques in the aortic tree of mice in the model group, while the plaque area in the BPQD group was significantly reduced by 45.3% compared to the model group. The effect was better than that of traditional statins (15.3% less than the model group). (Figure 8A). TEM images of the aorta showed numerous lipid droplets in the aortic wall in the model group, while lipid droplets reduced and autophagosomes appeared in the BPQD group (Figure 8C).

The results of the blood lipid test showed that the levels of total cholesterol (TC), triglyceride (TG), and low-density lipoprotein (LDL) in the model group increased, while those in the BPQD group decreased (Supplementary Figure 9A). The results of the serum inflammatory cytokine test showed that the levels of tumor necrosis factor-α (TNF-α), monocyte chemoattractant protein-1 (MCP-1) and interleukin 12 (IL12) in the model group increased, while those in the BPQD group decreased (Supplementary Figure 9B). Detection of the intestinal flora revealed that 2 bacterial communities (Enterobacteriaceae and Bacteroidaceae) in the model group disappeared, while that in the BPQD group reappeared to some extent (Supplementary Figure 10).

The results of the *in vivo* and *in vitro* experiments indicated that BPQDs cleared the aortic plaque area and restored partial vascular elasticity, thereby alleviating atherosclerosis. BPQDs may also promote autophagy of foam cells in the aortic wall. BPQDs also reduced liver fat accumulation, blood lipid levels, and serum inflammatory cytokines.

The recovery of vascular elasticity after treatment is a crucial index. The detection of vascular elasticity by photoacoustic microscopy is complementary to the detection of vascular elasticity by ultrasonography, but with the ability to provide additional microscopic treatment information. Photoacoustic imaging is a new and popular research field, representing an upcoming biomedical imaging modality availing the benefits of optical resolution and acoustic depth of penetration. Generally, PAI systems can be grouped into three configurations according to their combination of optical illumination methods and acoustic detection methods: tomography, mesoscopy, and microscopy systems [41]. Photoacoustic computed tomography (PACT) has been extensively used in human studies due to its deep tissue penetration (several centimeters), while photoacoustic microscopy offers higher resolution at the expense of penetration depth, which can also be advantageous in clinics [42, 43]. Owing to its excellent resolution, photoacoustic microscopes can detect changes in the inner diameter of blood vessels at the micron level, which has great advantages over traditional ultrasonic imaging. In the *in vivo* experiments, a photoacoustic microscope was used to detect subtle fluctuations in the internal diameter of the abdominal aorta in living mice and calculate the vascular elasticity of the abdominal aorta.

In this study, we found a positive effect of BPQDs in the treatment of atherosclerosis in mice, where they reduced atherosclerotic plaques and restored partial vascular elasticity. We demonstrated the safe and rapid removal effect of black phosphorus quantum dots on atherosclerotic plaque, finding it to be significantly better than that of simvastatin in clinical use. A positive control drug to allow comparisons between the test nanomaterial and well-known drugs has been reported in another research paper by our group [44]. Additionally, we found that BPQDs had a regulatory effect on the intestinal flora of mice. Two bacterial communities that disappeared in the atherosclerotic mice model, Bacteroidetes and Actinobacteria, reappeared after BPQD treatment, suggesting that they may be related to the development of atherosclerosis. The relationship between intestinal flora and atherosclerosis is worthy of further study.

The possible mechanism of BPQDs in the treatment of atherosclerosis was further explored in *in vitro* cell experiments, in which macrophages were used to conduct a series of experiments to preliminarily explore the effect and mechanism of BPQDs on macrophages, including reducing macrophage ROS, regulating macrophage polarization, and regulating macrophage lipid metabolism. We have preliminarily explored the possible mechanism of BPQDs in the treatment of atherosclerosis *in vitro*, although the targeting and mechanisms of BPQDs *in vivo* and their effects on cell signaling pathway regulation remain to be further studied in the future.

### Statistical analysis

IBM SPSS 26.0 and GraphPad Prism 5 were used for statistical analysis. Excel, ImageJ, Origin, and Photoshop were used to draw the images. The sample data obtained in the experiment were averaged three times and expressed as mean ±standard deviation. Comparisons between two groups were made using a t-test or one-way analysis of variance. Differences with P<0.05 (two-sided) were considered statistically significant.

## CONCLUSIONS

In this study, BPQDs were first applied to the treatment of atherosclerosis in high fat diet ApoE^-/-^ model mice that BPQDs were given every other day for 3 weeks without changing the high-fat diet. 45.3% atherosclerotic plaque was cleared efficiently within 3 weeks in BPQDs intravenous administration way every other day. The treatment was more effective than traditional statins. Moreover, BPQDs could restore part of arterial vascular elasticity, and reduce the levels of blood lipid indexes and inflammatory factors. We preliminarily explored the ways in which BPQDs alleviate atherosclerosis, mainly through the following possible interaction mechanisms (Figure 1): (1) reducing ROS and oxidative stress response; (2) inhibiting M1 macrophage polarization and promote M2 polarization, so as to play an anti-inflammatory role; (3) inhibiting the uptake of ox-LDL by macrophages and thus reducing the formation of foam cells; (4) promoting the autophagy of foam cells, thus reducing their accumulation in blood vessel walls; and (5) restoring normal intestinal flora.

Atherosclerosis is the pathological basis of cardiovascular disease, and there are no clinical drugs that can safely and efficiently remove atherosclerotic plaques. This study suggests that BPQDs may be a promising method for rapid treatment of atherosclerosis without changing dietary habits. The significance of drug development for the rapid treatment of chronic atherosclerosis is that it can greatly reduce the incidence of atherosclerosis-related diseases such as myocardial infarction and cerebral infarction without changing the dietary habits of patients, so that patients can get rid of the problems of liver and kidney function decline caused by long-term medication, and greatly improve the quality of life of patients.

## Supplementary Material

Supplementary Figures

Supplementary Tables
