# Supplementary material for: Study on the use of black phosphorus quantum dots in the treatment of atherosclerosis
Source: Aging (Albany NY). 2025 Feb 20;17(2):563–87. doi: 10.18632/aging.206205 (PMC11892921; doi:10.18632/aging.206205)
Supplement: Supplementary Figures [file aging-17-206205-s001.pdf]

SUPPLEMENTARY FIGURES

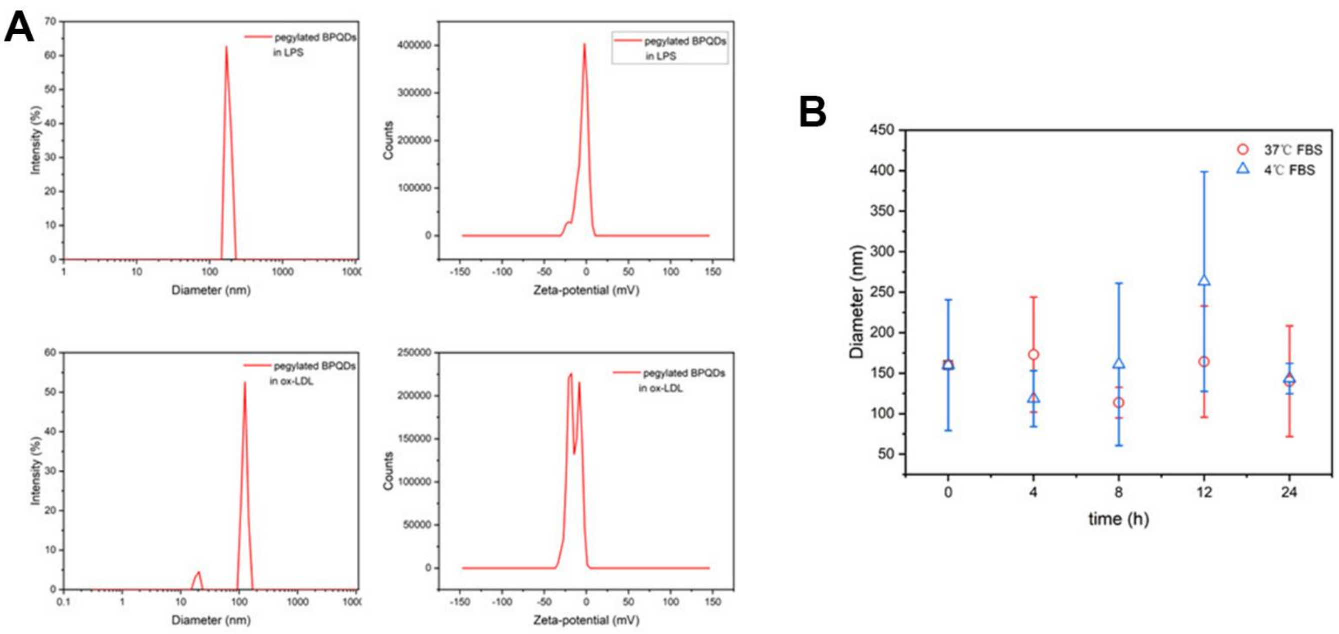

Supplementary Figure 1. (**A**) Particle size and potential of pegylated BPQDs in LPS and ox-LDL solutions. (**B**) Colloidal stability of pegylated BPQDs at different temperatures.

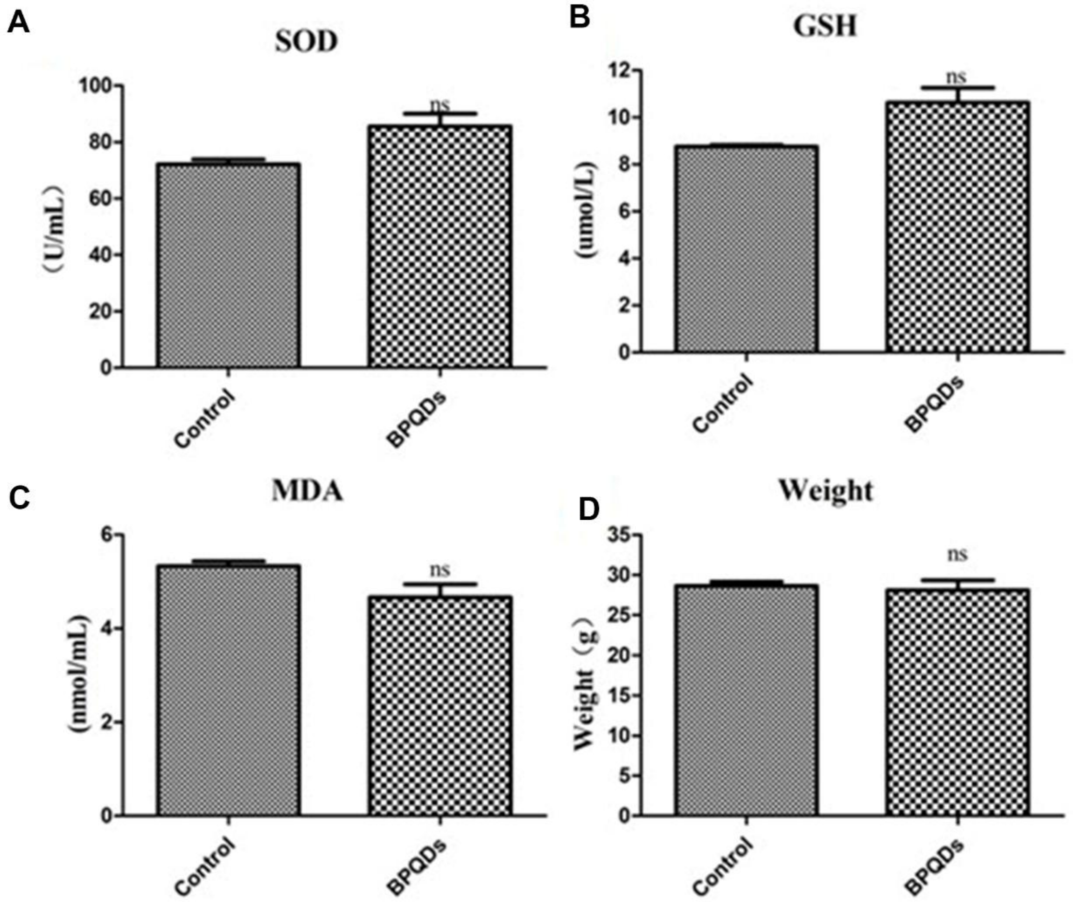

Supplementary Figure 2. (**A**–**C**) Serum oxidation factor levels in mice after 3 weeks of administration. (**D**) Body weight in mice after 3 weeks of administration. (SOD: Superoxide dismutase, GSH: Glutathione, MDA: Malondialdehyde) (ns: No significant difference compared to the control group.)

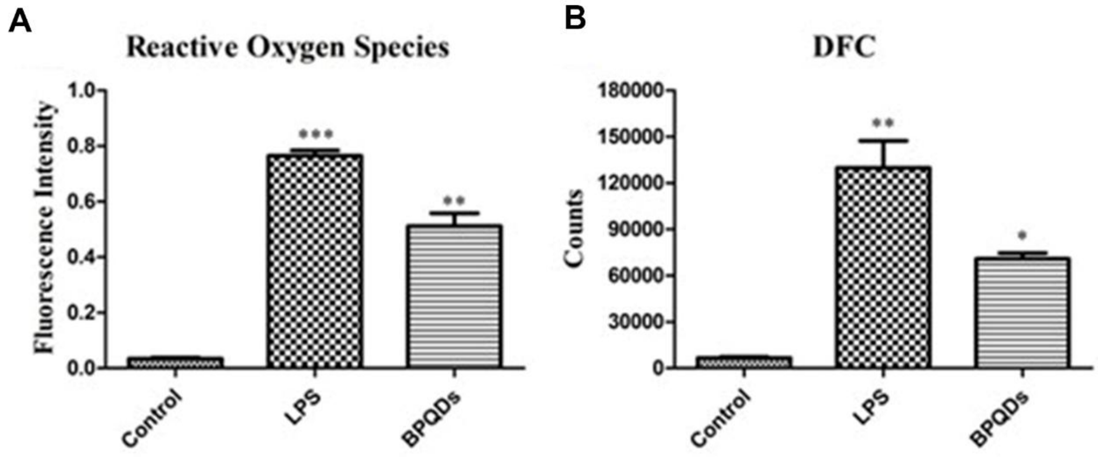

Supplementary Figure 3. (**A**) Statistical analysis of Figure 4A. (**B**) Statistical analysis of Figure 4B. (*: $P < 0.05$, **: $P < 0.01$, ***: $P < 0.001$ represent significant difference. LPS group compared to the control group, BPQDs group compared to the LPS group.)

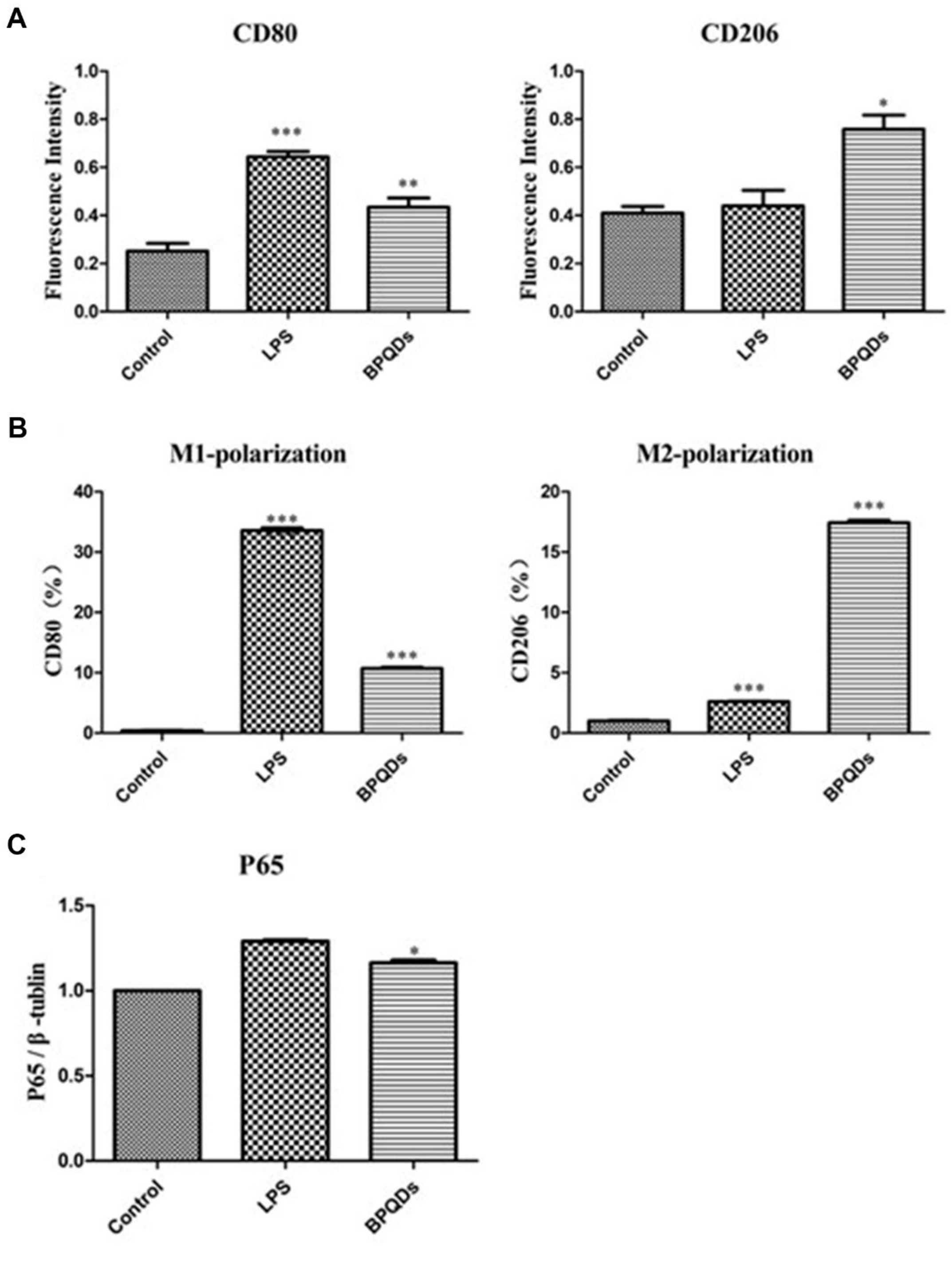

Supplementary Figure 4. (**A**) Statistical analysis of Figure 5A. (**B**) Statistical analysis of Figure 5B. (**C**) Statistical analysis of Figure 5C. (*: P < 0.05, **: P < 0.01, ***: P < 0.001 represent significant difference. LPS group compared to the control group, BPQDs group compared to the LPS group).

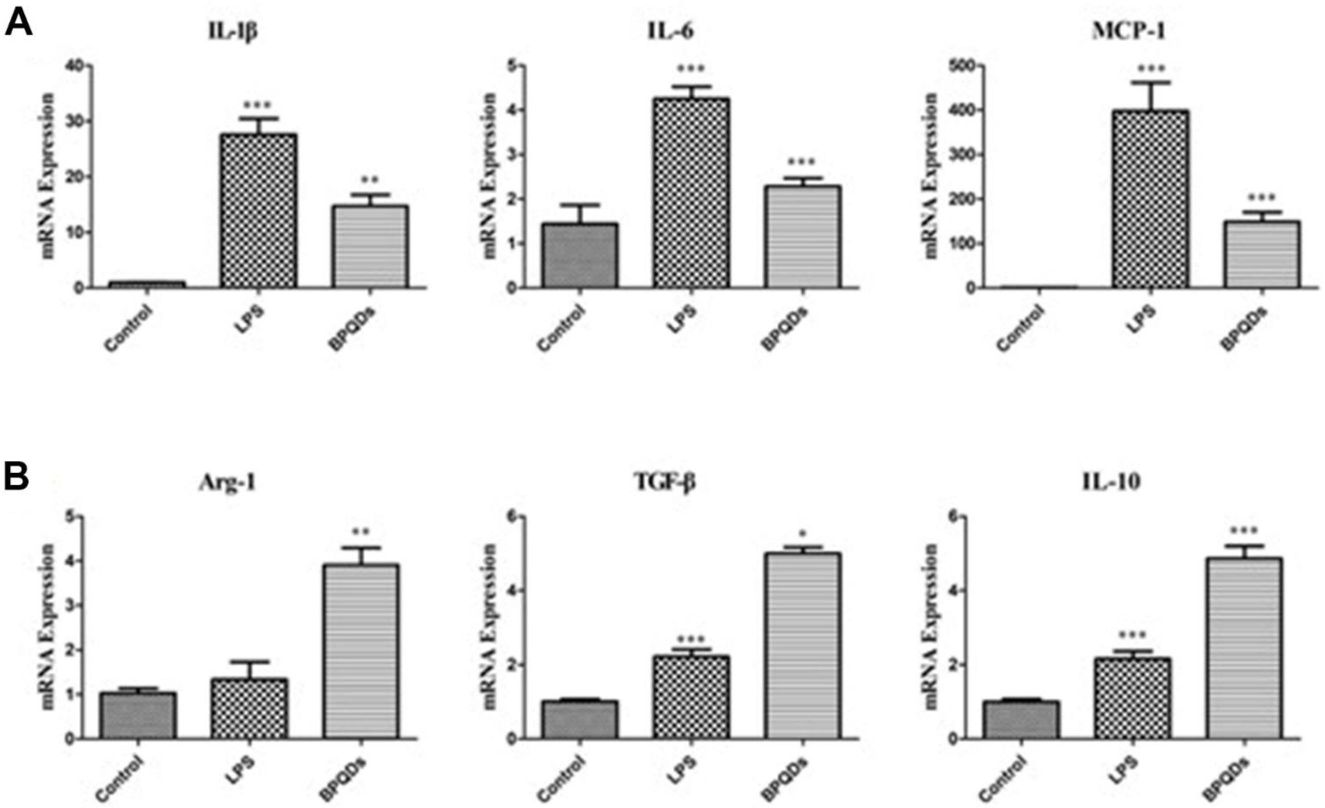

Supplementary Figure 5. (**A**) Gene expression levels of pro-inflammatory cytokines and chemokines. (**B**) Gene expression levels of inhibitory cytokines. (*: P < 0.05, **: P < 0.01, ***: P < 0.001 represent significant difference. LPS group compared to the control group, BPQDs group compared to the LPS group).

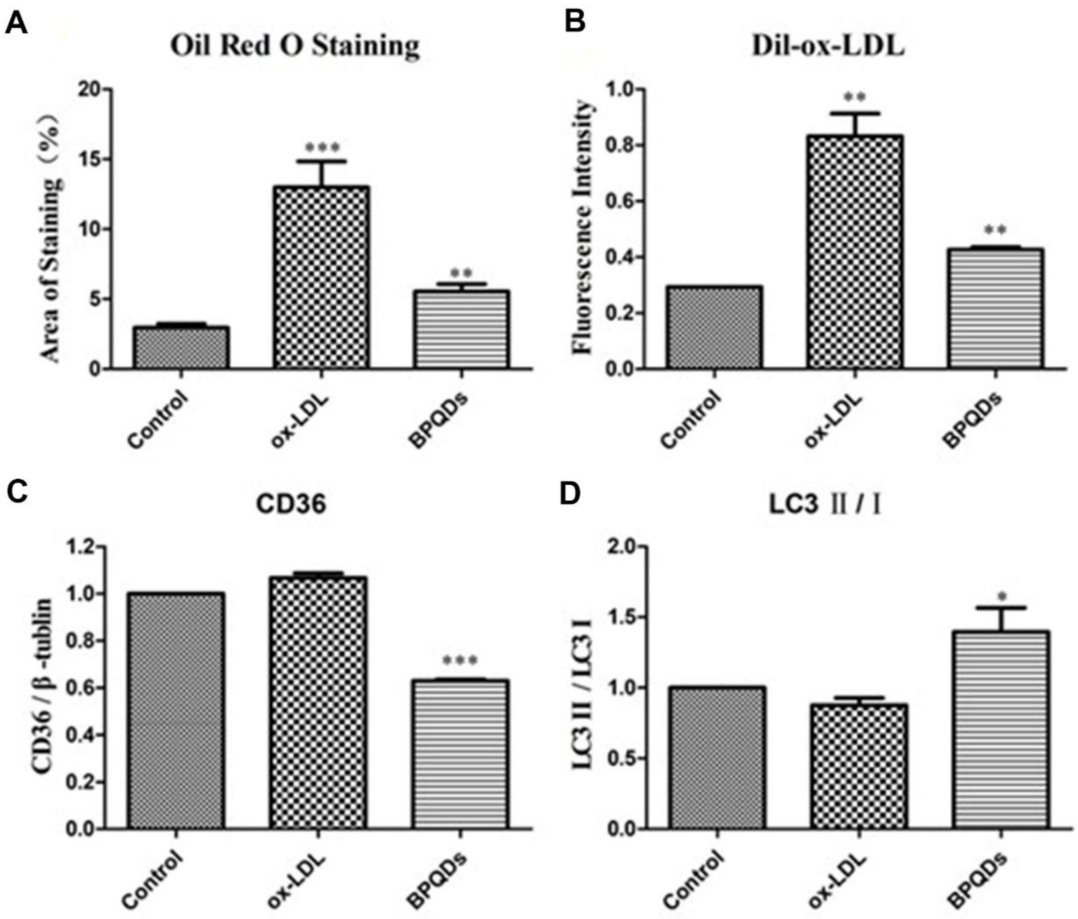

Supplementary Figure 6. (**A**) Statistical analysis of Figure 6A. (**B**) Statistical analysis of Figure 6B. (**C**, **D**) Statistical analysis of Figure 6C. (*: $P < 0.05$, **: $P < 0.01$, ***: $P < 0.001$ represent significant difference. Ox-LDL group compared to the control group, BPQDs group compared to ox-LDL group.)

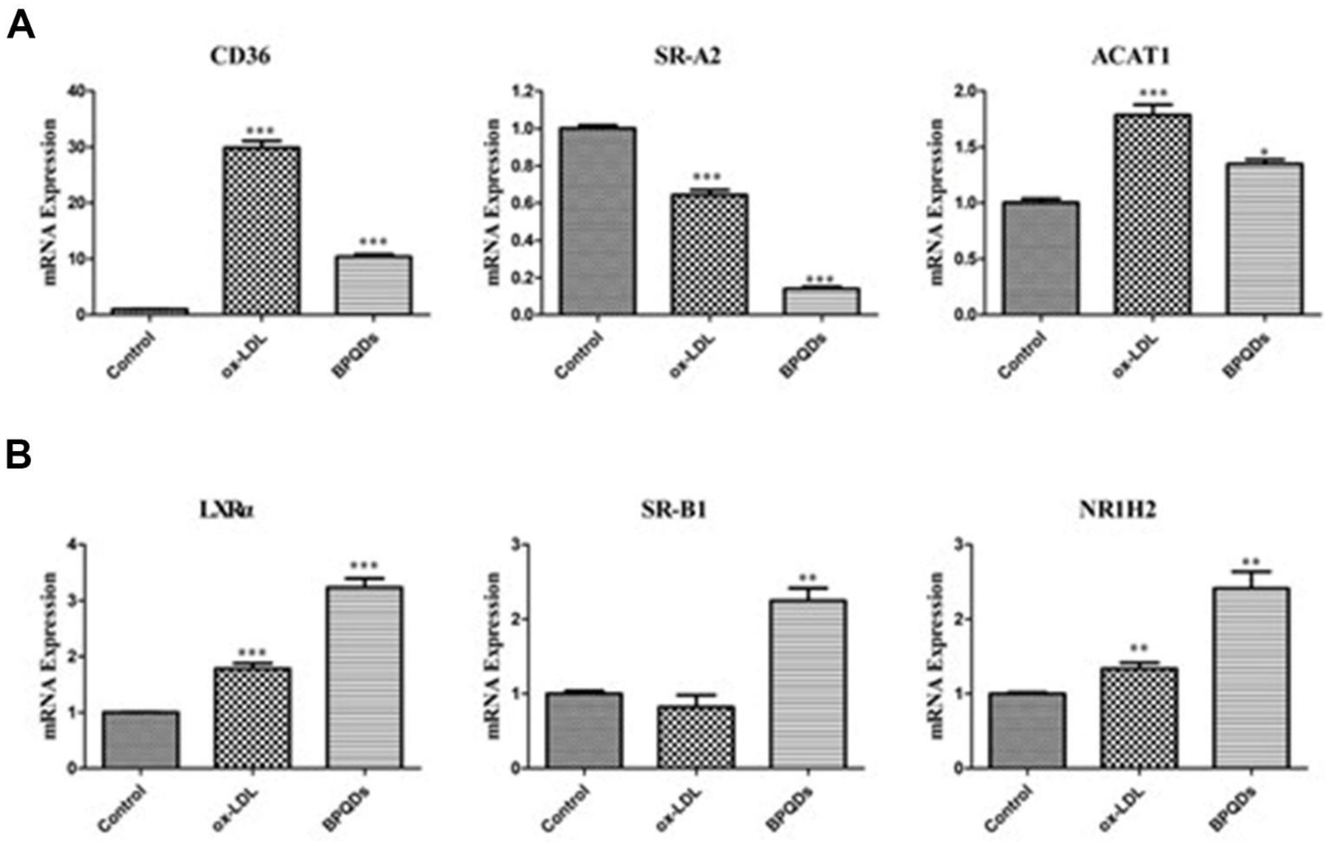

Supplementary Figure 7. (**A**) Gene expression levels of factors that promote the entry of lipids into macrophages or inhibit the lipid metabolism of macrophages. (**B**) Factors that inhibit the entry of lipids into macrophages or promote the lipid metabolism of macrophages. (*: P < 0.05, **: P < 0.01, ***: P < 0.001 represent significant difference. Ox-LDL group compared to the control group, BPQDs group compared to the ox-LDL group.)

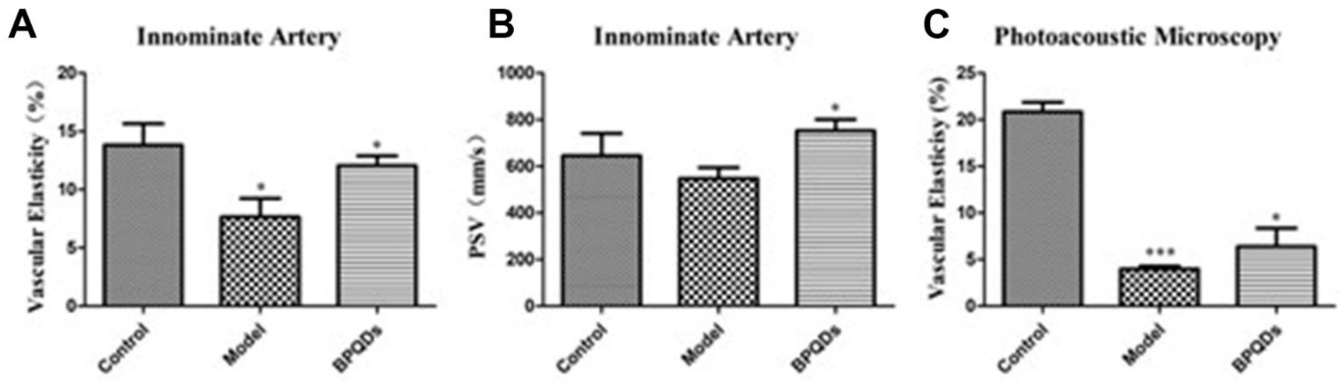

Supplementary Figure 8. (**A**) Vascular elasticity of the innominate artery. (**B**) Peak systolic velocity of the innominate artery. (**C**) Vascular elasticity of the abdominal artery under photoacoustic microscopy. (*: P < 0.05, **: P < 0.01, ***: P < 0.001 represent significant difference. Model group compared to the control group, BPQDs group compared to the model group.)

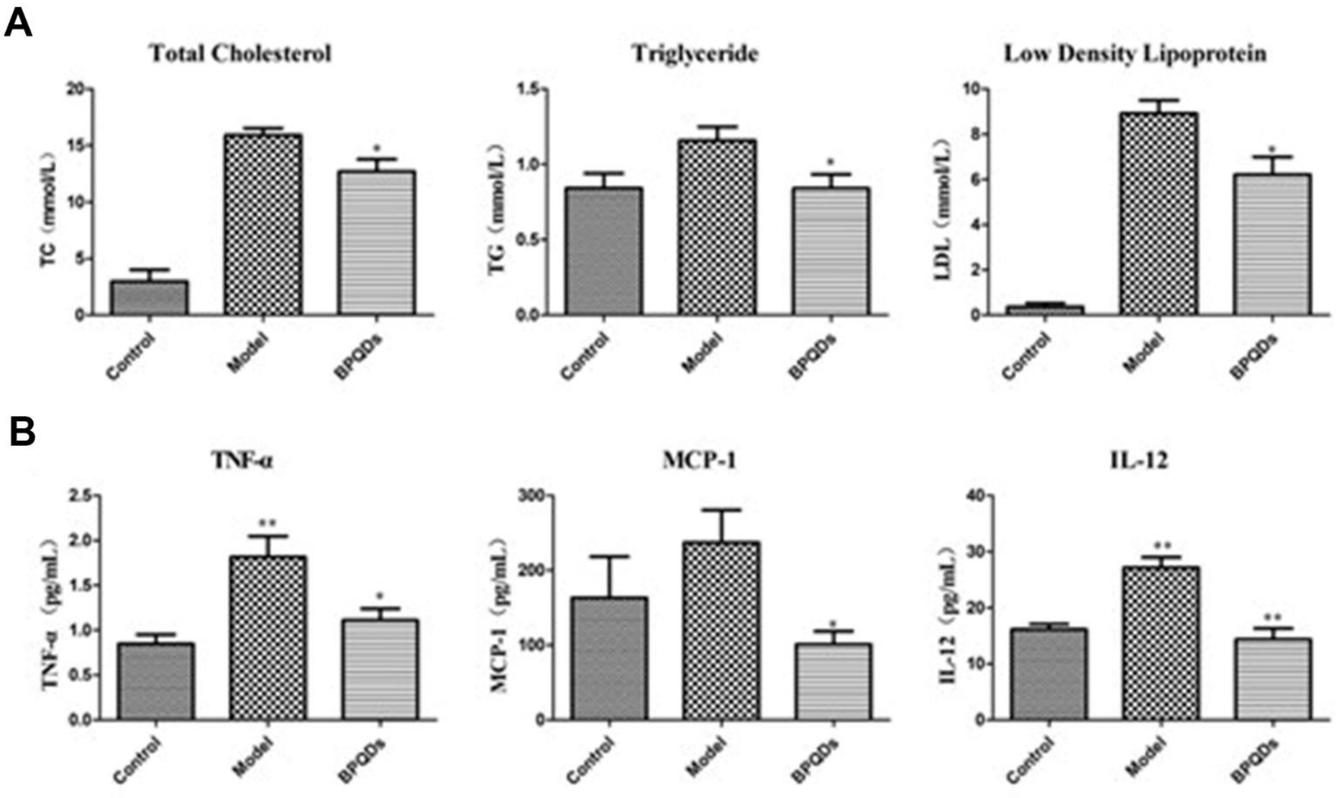

Supplementary Figure 9. (**A**) Levels of blood lipids. (**B**) Levels of serum inflammatory cytokines. (TNF-α: tumor necrosis factor-α, MCP-1: monocyte chemoattractant protein-1, IL-12: interleukin 12) (*: P < 0.05, **: P < 0.01 represent significant difference. Model group compared to the control group, BPQDs group compared to the model group.)

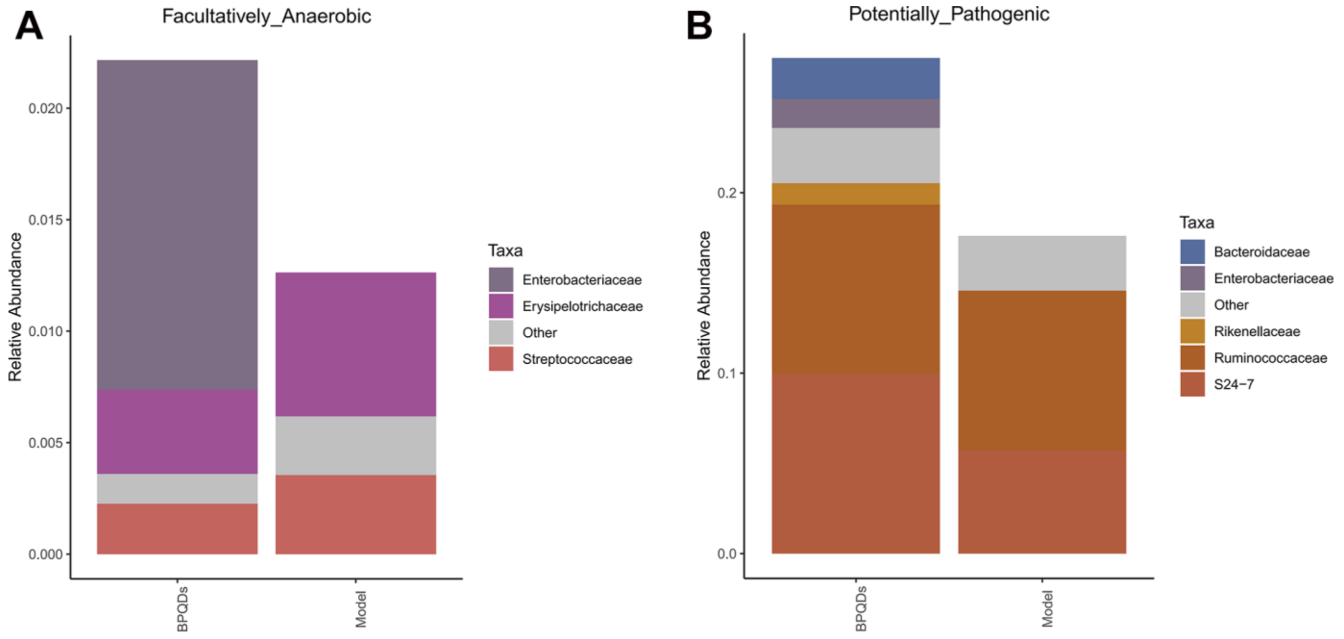

Supplementary Figure 10. (**A**, **B**) Changes of intestinal flora in mice. Enterobacteriaceae and Bacteroidaceae disappeared in the model group, and reappeared after BPQDs treatment.
