# Supplementary material for: Study on the use of black phosphorus quantum dots in the treatment of atherosclerosis
Source: Aging (Albany NY). 2025 Feb 20;17(2):563–87. doi: 10.18632/aging.206205 (PMC11892921; doi:10.18632/aging.206205)
Supplement: Supplementary Tables [file aging-17-206205-s002.pdf]

SUPPLEMENTARY TABLES

Supplementary Table 1. Experimental materials and sources.

A. Experimental mice

Strain	Age	Body weight	Sources
C57BL / Apoe$^{-/-}$	5 weeks	15-20g	Guangdong Pharmaceutical Kang Co.

B. Experimental cells

Cell tape	Sources
RAW264.7	Servicebio, Inc.
MAEC	Servicebio, Inc.

Supplementary Table 2. *In vivo* safety: grouping and administration of mice.

Group	Mice (n ≥ 5)	Dosage	Frequency
Control	C57BL	0.1 mL normal saline	3 times/week
BPQDs	C57BL	0.1 mL BPQDs in normal saline solution	3 times/week

Supplementary Table 3. Model and parameters of photoacoustic microscopes.

Model	VIS-H-50, PAOMTek, China
Laser output wavelength (nm)	523
Imaging range (mm)	3
Imaging speed (s/Volume)	5
Lateral resolution (μm)	4.6
Axial resolution (μm)	158
Applicable targets	Mouse, Rat, Rabbit, Human
Driving mode	Four-way differential drive
Operating ambient temperature	15-35° C
Imaging probe size	22*30*13
